# From ‘strong recommendation’ to practice: A pre-test post-test study examining adherence to stroke guidelines for fever, hyperglycaemia, and swallowing (FeSS) management post-stroke

**DOI:** 10.1016/j.ijnsa.2024.100248

**Published:** 2024-10-23

**Authors:** Kelly Coughlan, Tara Purvis, Monique F. Kilkenny, Dominique A. Cadilhac, Oyebola Fasugba, Simeon Dale, Kelvin Hill, Megan Reyneke, Elizabeth McInnes, Benjamin McElduff, Jeremy M. Grimshaw, N Wah Cheung, Christopher Levi, Catherine D'Este, Sandy Middleton

**Affiliations:** a Nursing Research Institute, St Vincent's Health Network Sydney, St Vincent's Hospital Melbourne and Australian Catholic University, Level 5, deLacy Building, 390 Victoria Street, Darlinghurst, NSW 2010, Australia; b School of Nursing, Midwifery and Paramedicine, Australian Catholic University, 33 Berry Street, North Sydney, NSW 2060, Australia; c Sroke and Ageing Research, School of Clinical Sciences, Monash University. Monash Medical Centre, Block E, Level 5, 246 Clayton Rd, Clayton, VIC 3168, Australia; d Stroke Theme, The Florey Institute of Neuroscience and Mental Health, University of Melbourne, 245 Burgundy Street, Heidelberg, VIC 3084, Australia; e Stroke Foundation, Level 7/461 Bourke St, Melbourne, VIC 3000, Australia; f Ottawa Health Research Institute, Ottawa Hospital - General Campus, Centre for Practice-Changing Research (CPCR); and University of Ottawa, 501 Smyth Box 511, Ottawa, ON K1H 8L6, Canada; g Centre for Diabetes and Endocrinology Research, Westmead Hospital and University of Sydney, Hawkesbury Road, Westmead, NSW 2145, Australia; h John Hunter Hospital, University of Newcastle. Lookout Rd, New Lambton Heights, NSW 2305, Australia; i Sax Institute, Level 3/30C Wentworth St, Glebe, NSW 2037, Australia; j School of Medicine and Public Health, College of Health, Medicine and Wellbeing, University of Newcastle, Newcastle, NSW, Australia

**Keywords:** Stroke, Guidelines, Implementation, Fever, Hyperglycaemia, Swallow, Sustainability

## Abstract

**Background:**

The Quality in Acute Stroke Care (QASC) Trial demonstrated that assistance to implement protocols to manage Fever, hyperglycaemia (Sugar) and Swallowing (FeSS) post-stroke reduced death and disability. In 2017, a ‘Strong Recommendation’ for use of FeSS Protocols was included in the Australian Clinical Guidelines for Stroke Management. We aimed to: i) compare adherence to FeSS Protocols pre- and post-guideline inclusion; ii) determine if adherence varied with prior participation in a treatment arm of a FeSS Intervention study, or receiving treatment in a stroke unit; and compare findings with our previous studies.

**Methods:**

Pre-test post-test study using Australian acute stroke service audit data comparing 2015/2017 (pre-guideline) versus 2019/2021 (post-guideline) adherence. Primary outcome was adherence to all six FeSS indicators (composite), with mixed-effects logistic regression adjusting for age, sex, stroke type and severity (ability to walk on admission), stroke unit care, hospital prior participation in a FeSS Intervention study, and correlation of outcomes within hospital. Additional analysis examined interaction effects.

**Results:**

Overall, 112 hospitals contributed data to ≥1 one Audit cycle for both periods (pre=7011, post=7195 cases); 42 hospitals had participated in any treatment arm of a FeSS Intervention study. Adherence to FeSS Protocols post-guideline increased (pre: composite measure 35% vs post: composite measure 40 %, aOR:1.2 95 %CI: 1.2, 1.3). Prior participation in a FeSS Intervention study (aOR:1.6, 95 %CI: 1.2, 2.0) and stroke unit care (aOR 2.3, 95 %CI: 2.0, 2.5) were independently associated with greater adherence to FeSS Protocols. There was no change in adherence over time based on prior participation in a FeSS Intervention study (*p* = 0.93 interaction), or stroke unit care (*p* = 0.07 interaction).

**Conclusions:**

There is evidence of improved adherence to FeSS Protocols following a ‘strong recommendation’ for their use in the Australian stroke guidelines. Change in adherence was similar independent of hospital prior participation in a FeSS Intervention study, or stroke unit care. However, maintenance of higher pre-guideline adherence for hospitals prior participation in a FeSS Intervention study suggests that research participation can facilitate greater guideline adherence; and confirms superior care received in stroke units. Nevertheless, less than half of Australian patients are being cared for according to the FeSS Protocols, providing impetus for additional strategies to increase uptake.

**Contributions to the literature**

**What is already known**

Inclusion in the national stroke clinical practice guidelines came several years after seminal trial results were published.

Sustained practice change after clinical trial completion and efforts to upscale and disseminate results are rarely studied longer term.

**What this paper adds**

The overall improved adherence to FeSS Protocols following their inclusion in the guidelines is likely influenced by multiple factors.

Sustained adoption of clinical guidelines in Australian hospitals is further enhanced by specialist stroke unit care and participation in implementation studies where external support is provided.

This evaluation of a complex health intervention at the population level will assist with future implementation and dissemination efforts to maximise national adoption of FeSS Protocols

Alt-text: Unlabelled box

## 1 Background

Facilitated implementation of a nurse-led intervention to manage **Fe**ver, hyperglycaemia (**S**ugar) and **S**wallowing (**FeSS** Protocols) in stroke units was shown to reduce death and disability (15.7 % adjusted absolute difference) at 90 days post-stroke for patients in the cluster randomised Quality in Acute Stroke Care (QASC) Trial (Middleton et al., 2011). This effect was sustained, with over 20 % of patients more likely to be alive four years following their stroke (absolute risk reduction 5 %) (Middleton et al., 2017). Subsequent statewide scale-up of the protocols in the pre-test post-test Quality in Acute Stroke Care Implementation Project (QASCIP) during 2013–2014 demonstrated improvements in protocol adherence across New South Wales, Australia (Middleton et al., 2016). The effectiveness of the FeSS Protocols in the emergency department setting in the Triage, Treatment, and Transfer (T^3^) cluster randomised Trial was later evaluated between 2013 and 2016. However, uptake by clinicians within emergency departments was poor with no differences found between treatment and control groups in process measures or patient outcomes (Middleton et al., 2019).

In 2017, a ‘Strong Recommendation’ to support use of the FeSS Protocols was included in the Australian Clinical Guidelines for Stroke Management (hereafter referred to as the Stroke Guidelines) based on the QASC Trial results, namely: ‘*All acute stroke services should implement standardised protocols to manage fever, glucose and swallowing difficulties in stroke patients* (Middleton et al., 2011)*’* (National Stroke Foundation. Clinical Guidelines for Stroke Management, 2017). Swallow processes of care have been included in the clinical guidelines since 2007; however this was the first time recommendations related to fever and hyperglycaemia protocols were included. ^5^

It is commonly cited that it takes 17 years on average for only 14 % of new scientific discoveries to enter day-to-day clinical practice (Morris et al., 2011; Balas and Boren, 2000). Outside of the FeSS Intervention studies (QASC, QASCIP and T^3^) that used multi-faceted implementation strategies (audit and feedback, clinical champions, barrier and enabler assessments, educational workshops and reminders) there has not been any systematic roll-out of the FeSS Protocols across Australia. Adherence to the FeSS Protocols has previously been evaluated using data from the National Stroke Audits (acute services) by Purvis et al. (2019) which demonstrated an increased uptake in their use over a 4-year period (2013–2017) (Purvis et al., 2019). However, a significant evidence-practice gap remained with only 41 % of patients receiving care in accordance with all FeSS Protocols in 2017. In that study, greater uptake of the FeSS Protocols was also reported for hospitals that participated in the two original FeSS Intervention studies, QASC and QASCIP, up to six years following the original trial (Purvis et al., 2019). This finding is in contrast to the literature that suggests adherence to clinical practice guidelines after an implementation research trial tends to decrease after one year (Ament et al., 2015).

Historically, the distribution of untargeted publication of clinical practice guidelines (i.e. passive dissemination) (Vedel et al., 2018) has been reported as ineffective at changing clinical practice when used in isolation (Prior et al., 2008). The improvements in healthcare professional behaviour changes are reported to be more effective when more active implementation and dissemination techniques (especially multi-faceted strategies) are employed (Prior et al., 2008). However, there is an argument that the significant costs and resources associated with some active implementation strategies (e.g. audit and feedback, educational meetings and outreach) potentially outweigh the benefits of the clinical practice guideline being introduced (Grimshaw et al., 2004). Less expensive strategies (e.g. dissemination educational materials and/or clinical practice guidelines) that are simpler to implement may also be more sustainable (Squires et al., 2014).

The inclusion of the new Stroke Guideline recommendation for use of the FeSS Protocols provided a unique opportunity to assess adherence to FeSS Protocols beyond 2017 and its impact on improved care delivery using the National Stroke audit (acute services) data.

We aimed to i) compare adherence to FeSS Protocols pre- and post-inclusion in Stroke Guidelines; ii) determine if changes in FeSS Protocol adherence varied based on a) hospital prior participation in any treatment arm of a FeSS Intervention study or b) receiving treatment in a stroke unit and; iii) compare adherence to individual FeSS indicators across FeSS Intervention studies.

## 2 Methods

### 2.1 Study design and setting

A pre-test post-test study was undertaken using retrospective clinical data from hospitals that participated in the biennial Australian National Stroke Audit (acute services) comparing 2015 and 2017 audit years (pre-guideline); with 2019 and 2021 audit years (post-guideline).

Established in 2007, the Australian National Stroke Audit (herein referred to as the Audit) aims to measure hospitals’ adherence to evidence-based practice recommendations as outlined in the Clinical Guidelines for Stroke Management (Stroke Foundation, 2020; Harris et al., 2010). The voluntary audit alternates between acute stroke services and inpatient rehabilitation services each year, and includes national representation (acute stroke services participation rate in 2019 and 2021: 76 % & 79 % of all eligible public services respectively) (Stroke Foundation 2019, Stroke Foundation, 2021). Hospitals must admit approximately 50 patients with stroke annually to be eligible.

### 2.2 The national stroke audit program

The methods for the Audit program have been detailed elsewhere (Stroke Foundation, 2020; Harris et al., 2010). Briefly, this involves: i) completion of a self-reported organisational survey by hospital clinicians that evaluates hospital adherence to the National Acute Stroke Services Framework (Stroke Foundation. National Acute Stroke Services Framework, 2019; National Stroke Foundation. National Acute Stroke Services Framework, 2015) (e.g. in-hospital stroke services and processes such as access to CT, acute therapies and stroke unit care) and ii) a retrospective clinical audit of approximately 40 consecutive acute stroke cases per hospital (recently increased to 60 cases for 2023 Audit cycle) (Stroke Foundation 2023). The in-hospital clinical care provided is measured against evidence-based Clinical Guidelines for Stroke Management (National Stroke Foundation. Clinical Guidelines for Stroke Management, 2017); and the Australian Commission of Safety and Quality in Health Care's Acute Stroke Clinical Care Standard (ACSQHC, 2019). The audit periods reflect all hospital admissions with stroke diagnosis from June of the previous year, for example, the 2017 Audit cycle reflects patient admissions with stroke from 1st June and discharged prior to 31st December 2016. Process of care indicators related to the management of fever, hyperglycaemia and swallowing during the in-hospital admission period for stroke have been included in the audit since 2013.

Auditors are trained in data abstraction and data entry into the Australian Stroke Data Tool (AuSDaT) platform, with inter-rater reliability cases provided from each site (Stroke Foundation, 2020). The AuSDaT is a national, online database platform that enables the standardised and systematic data collection for multiple stroke data collection programs (Australian Stroke Coalition, 2023; Ryan et al., 2022). This Australia-wide audit of clinical practice in acute stroke hospital services is designed to promote quality improvements in stroke care. The results are fed back in a tailored report to hospitals for them to identify areas for improvement.

### 2.3 Hospital and participant eligibility

Following approval processes, data were obtained from the Stroke Foundation for all Australian hospitals that participated in the 2015, 2017, 2019 and 2021 Audits. To be included in this analysis, hospitals had to have contributed data into at least one Audit cycle for both time periods (pre-guideline 2015 and/or 2017; and post-guideline 2019 and/or 2021). All patients with a primary diagnosis of stroke (ischaemic, haemorrhagic or undetermined) and aged ≥18 years were included. Patients who experienced a transient ischaemic attack (TIA) were excluded in addition to patients that were documented for palliative care measures (as this indicates the patient was not receiving curative treatments during their hospital admission, only pain relieving and comfort measures).

### 2.4 Data collection

**Fig. 1:**
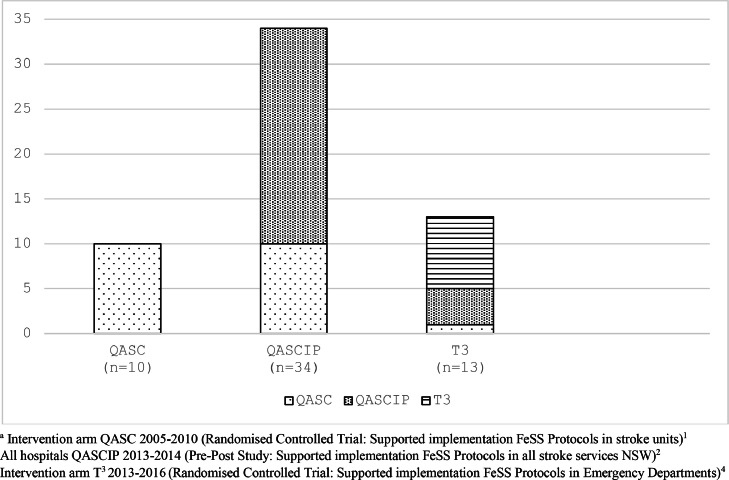
Hospital participation in treatment arm of FeSS Intervention studies.

The FeSS variables (Box 1) were extracted from audit data in addition to patient characteristics: age, sex, pre-morbid dependency level (modified Rankin scale[mRS]) (Banks and Marotta, 2007), prior risk factors, stroke type, and stroke severity (being able to walk independently on admission was used as a validated proxy for this measure) (Cadilhac et al., 2019; Counsell et al., 2002; Kilkenny et al., 2020). Treatment with thrombolysis, receipt of care in a stroke unit, discharge dependency level (mRS) and discharge destination were collected with minimal hospital organisational characteristics (presence of a stroke unit, protocols to manage fever, and/or hyperglycaemia and/or swallow). Hospitals that had previously participated in any treatment arm of a FeSS Intervention study were defined as: the intervention hospitals in the Quality in Acute Stroke Care [QASC] randomised controlled trial; and/or the intervention hospitals in Triage, Treatment, and Transfer [T^3^] randomised controlled trial (Middleton et al., 2019), and/or all hospitals in the Pre-Post QASC Implementation Project [QASCIP] (Middleton et al., 2016) (Fig. 1).

### 2.5 Outcome measures

The outcome was an overall binary process of care adherence measure that reflected whether or not the patient received all six processes of care elements of the FeSS protocol assessed as part of the audit (composite measure) (Box. 1). This measure of defect-free care used throughout the QASC research program (Middleton et al., 2011, 2016, 2019; Purvis et al., 2019; Middleton et al., 2022) was used to enable reliable comparisons with research that preceded the new Stroke Guideline inclusion. Consistent with how Audit data have previously been reported, clinical impairments (e.g. ability to walk) were incorporated if the response was valid. However, for process of care indicators, not documented and unknown responses were assumed to be negative and included in the denominator (Cadilhac et al., 2019; Purvis et al., 2023).

**Box. 1:**
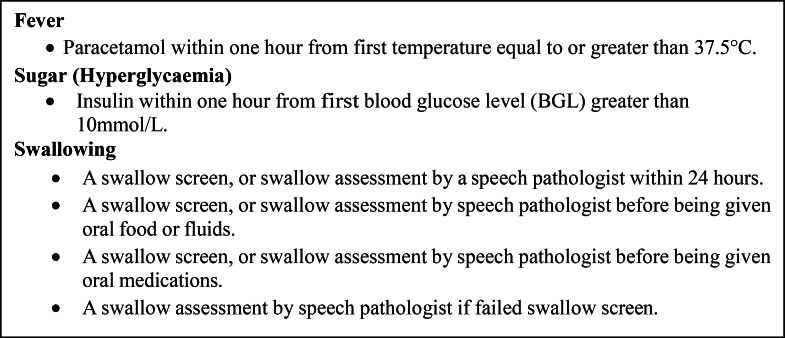
Adherence to FeSS Protocols composite measure comprised of six individual indicators.

### 2.6 Statistical analysis

Patient sociodemographic and clinical characteristics, and hospital characteristics are presented for audit data obtained from pre (2015 / 2017) and post (2019 / 2021) Stroke Guideline inclusion.

Mixed-effects logistic regression was undertaken to compare adherence to FeSS Protocols composite measure pre- versus post- Stroke Guideline inclusion, adjusting for age category (<65, 65–74, 75–84, 85+), sex, stroke type (haemorrhagic, ischaemic and undetermined) and severity (ability to walk on admission), hospital prior participation in a FeSS Intervention study, stroke unit care, and for correlation of outcomes within hospital. Regression models were also generated which included interaction terms (in addition to the previously specified covariates) to evaluate whether any pre-post Stroke Guideline inclusion changes in overall adherence to FeSS Protocols composite measure differed by: (i) hospital prior participation in any treatment arm of a FeSS Intervention study, and ii) treatment in a stroke unit and iii) each of these two factors.

The number and percentage of participants who had received each of the six FeSS indicators are reported for the pre- and post- Stroke Guideline inclusion samples. Mixed-effects logistic regression was undertaken to compare if any differences in these groups reached statistical significance using the previously specified covariates. Adjusted odds ratios (aOR) and 95 % confidence intervals are reported from logistic regression models. Sensitivity analyses were undertaken comparing 2017 and with the last Audit cycle (2021) to determine if pooling data from the Audit cycles had an effect on the outcome.

To situate real-world clinical practice findings from the Audits, in the broader context of this study, the number and percentage of participants who had received each of the six FeSS indicators are reported displayed from the pre- and post- guideline Audit data compared to the post-intervention results from the FeSS Intervention studies (QASC 2010, QASCIP 2014, T3 2016). Analyses was performed using Stata SE 15.0(www.stata.com) and R statistical software (R Core Team, 2023).

### 2.7 Ethics approval and consent to participate

Ethics approval for the study has been obtained from the Australian Catholic University Human Research Ethics Committee (2021–297 N). External approvals from the Stroke Foundation and Monash University (35037) were also provided. Individual patient consent was not required for this study as the data were not identifiable at the individual level, with summary data reported. The protocol for this study was registered at the Australia New Zealand Clinical Trials registry on the 1 May 2023 (CTRN 12623000445673).

## 3 Results

A total of 16,345 records from 131 hospitals were provided from the four Audit cycles. We excluded data from 19 hospitals (involving 2139 patient records (13 %) since these hospitals had not contributed audit data for at least one cycle for either the pre-or post-guideline study periods. Patient records with the primary intent of treatment being palliative care or with a TIA diagnosis, were also excluded.

**Fig. 2:**
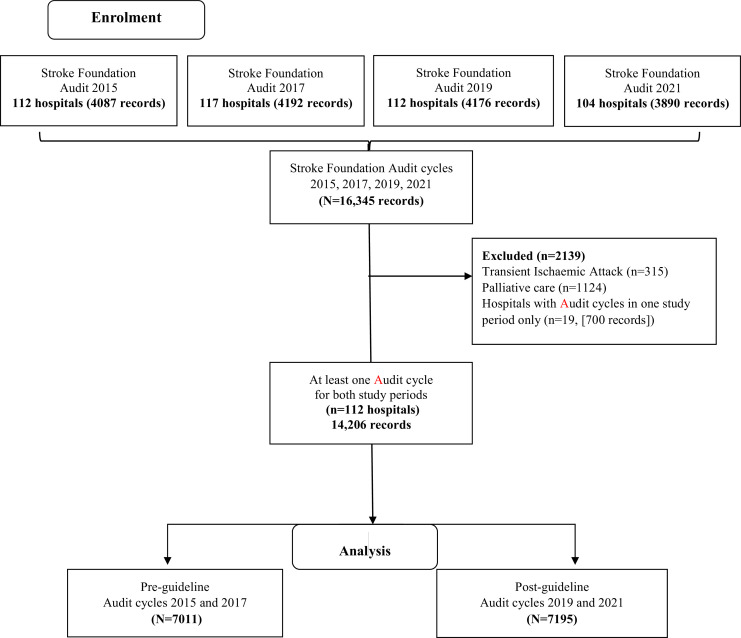
FeSS Adherence flow diagram.

Therefore, 112 hospitals were included in the final dataset: pre-guideline (2015, 2017 pooled Audit cycle data [*n* = 7011 patient records]), post-guideline (2019, 2021 pooled Audit cycle data [*n* = 7195 patient records]) (Fig. 2). Of these 112 hospitals, 42 had participated in the treatment arm of one or more FeSS Intervention studies QASC, QASCIP and/or T^3^ (Fig. 1).

### 3.1 Participant and hospital characteristics

Patient demographics were similar between the pre-guideline and post-guideline groups for age, sex and pre-morbid level of dependence (mRS 0–2) (Table 1). The post-guideline cohort had more ischaemic strokes (pre-guideline: 83 %, post-guideline: 86 %, *p* < 0.001), but fewer strokes that were classified as undetermined (pre-guideline: 6 %, post-guideline: 3 %, *p* *<* 0.001). These differences, while minor and clinically not significant, were likely due to neuroimaging advances and increased accessibility (e.g. change from clinical to tissue-based diagnosis for TIA's and mild strokes).

**Table 1:** Patient characteristics pre- and post-guideline inclusion period.

	Pre-guideline (*N* = 7011) n (%)	Post-guideline (*N* = 7195) n (%)	*p-*value^a^
PATIENT CHARACTERISTICS			
Age <65 y	1796 (26 %) b	1862 (26 %)	0.186
Age 65–74 y	1732 (25 %) b	1820 (25 %)	
Age 75–84 y	2049 (29 %) b	2152 (30 %)	
Age ≥85 y	1426 (20 %) b	1361(19 %)	
Sex, male	3969 (57 %)^b^	4177 (58 %) b	0.080
Independence prior to stroke (mRS 0–2)	5835 (83 %)	6025 (84 %)	0.411
Atrial fibrillation	1671 (27 %) c	1624 (24 %) d	**<0.001**
Previous Stroke	1476 (23 %) e	1524 (22 %) d	0.064
Ischaemic Heart Disease	1648 (27 %) c	1560 (23 %) d	**<0.001**
**CLINICAL CHARACTERISTICS**			
Ischaemic Stroke	5807 (83 %)	6198 (86 %)	**<0.001**
Haemorrhagic Stroke	775 (11 %)	760 (11 %)	0.346
Undetermined Stroke	429 (6 %)	237 (3 %)	**<0.001**
Walk independently on admission	3203 (47 %) d	3215 (46 %) d	0.146
Thrombolysis (ischaemic stroke)	583 (10 %)	684 (11 %)	0.076
Screened for thrombolysis	2931 (42 %)	4242 (59 %)	**<0.001**
Stroke unit care (if hospital had a stroke unit)	5037 (83 %)	5332 (85 %)	**0.002**
**ORGANISATIONAL CHARACTERISTICS**			
	**Pre-guideline** **(*N*=112)** n (%)	**Post-guideline** **(*N*=112)** n (%)	***p* value**^a^
Hospitals with a stroke unit	90/112 (80 %)	94/112 (84 %)	0.601
Hospital had Fever Protocols	104/112 (93 %)	107/112 (96 %)	0.569
Hospital had Sugar Protocols	101/112 (90 %)	105/112 (94 %)	0. 462
Hospital had Swallow Protocols	110/112 (98 %)	110/112 (98 %)	1.0

a chi-square test, mRS: Modified Rankin Scale; numbers may not add to total sample size due to missing values

b <1 % unknown/missing responses excluded

c 11–12 % unknown/missing responses excluded.

d 1–5 % unknown/missing responses excluded.

e 6–10 % unknown/missing responses excluded.

There was no difference between the pre-and post- Stroke Guideline inclusion groups for history of previous stroke, but there were more patients in the pre-guideline group with documented atrial fibrillation (pre-guideline: 27 %; post-guideline 24 %, *p* < 0.001) and ischaemic heart disease (pre-guideline: 27 % post-guideline: 23 %, *p* < 0.001). Although comorbidities are associated with greater disability and increased stroke severity, the variables used to evaluate these characteristics showed no differences between the pre- and post-guideline groups: independence prior to stroke (mRS 0–2) and being able to walk on admission (validated stroke severity proxy).

Although more patients were screened for possible treatment with thrombolysis in the post-guideline group (pre-guideline: 42 %, post-guideline: 59 %, *p* < 0.001) there was no difference in the proportion of patients who subsequently received intravenous thrombolysis treatment between the pre- and post-guideline groups.

The small increase in the number of hospitals that reported a dedicated stroke unit was not statistically significant but may have contributed to the increased number of patients able to access stroke unit care (pre-guideline: 83 %, post-guideline: 85 %; *p* = 0.002). The marginal increase in the number of hospitals that reported the presence of locally agreed management (including assessment/monitoring) protocols for fever and hyperglycaemia after the guideline introduction also did not reach statistical significance (Table 1) but are an important indicator of organisational readiness: Fever Protocols pre-guideline:93 %, post-guideline 96 %; Sugar Protocols pre-guideline: 90 %, post-guideline 94 %.

### 3.2 Overall adherence to FeSS Protocols post inclusion in Stroke Guidelines

As interaction terms were not statistically significant at the 5 % level, α = 0.05, (indicating that pre- to post- Stroke Guideline inclusion, changes in adherence to FeSS Protocols did not differ by hospital prior participation in the treatment arm of a FeSS Intervention study (*p* *=* 0.93*)* or treatment in a stroke unit (*p* = 0.07) (Supplemental Table 1), results are therefore presented for the main effects model only.

In the adjusted model, compared to the pre-guideline group, there was a significant improvement in overall adherence to FeSS Protocols for the post-guideline group: pre-guideline adherence to FeSS Protocols composite measure 2449/7011 (35 %); post-guideline 2864/7195 (40 %) aOR 1.2, 95 % CI 1.2, 1.3, *p* < 0.001 (Table 2). Patients aged ≥ 85 years had statistically significant greater odds of being treated according to FeSS Protocols compared to younger age categories: aOR 1.3, 95 % CI 1.1, 1.4, *p* < 0.001. However, patients with haemorrhagic stroke had lower odds of being treated according to the FeSS Protocols: aOR 0.74, 95 % CI 0.66, 0.84, *p* < 0.001. Similar findings were noted for patients who were able to walk on admission (indicating a mild stroke presentation): aOR 0.81, 95 % CI 0.75, 0.88, *p* < 0.001 (Table 2).

**Table 2:** Logistic Regression Model: Factors associated with adherence to FeSS Protocols composite measure.

	aOR^b^	95 % CI	*p*-value
Post-guideline period^a^	1.2	1.2, 1.3	**<0.001**
Hospital participated in treatment arm FeSS Intervention study	1.6	1.2, 2.0	**<0.001**
Received stroke unit care	2.3	2.0, 2.5	**<0.001**
Age			
<65 y	ref		
65–74 y	1.1	0.97, 1.2	0.194
75–84 y	1.1	0.99, 1.2	0.090
≥85 y	1.3	1.1, 1.4	**<0.001**
Male	1.0	0.95, 1.1	0.450
Haemorrhagic stroke	0.74	0.66, 0.84	**<0.001**
Walk independently on admission	0.81	0.75, 0.88	**<0.001**

a Reference is pre-guideline period.

b adjusted for factors listed in table including correlation of outcomes within hospital aOR - adjusted odds ratio, CI - confidence interval.

### 3.3 Adherence to FeSS Protocols and previous participation in a FeSS Intervention study

Overall, regression analyses demonstrated that patients who were cared for in hospitals that had previously participated in any treatment arm of a FeSS Intervention study were significantly more likely to receive care according to the FeSS Protocols (aOR 1.6, 95 % CI 1.2, 2.0, *p* < 0.001) when compared to those cared for in hospitals that had not participated (or were not exposed to the intervention) in these studies (Table 2).

This is reflected in adherence to FeSS Protocols being higher both pre- and post-guidelines inclusion in hospitals that had participated in the treatment arm of a FeSS Intervention study (pre-guideline: exposed 40 %, unexposed 31 %, post-guideline: exposed 46 %, unexposed 35 %, *p*<0.001). However, irrespective of exposure to treatment arm of a FeSS intervention, the change in adherence between pre- and post-guidelines was similar (*p*-value for interaction term *p* = 0.93) (Supplemental Table I).

### 3.4 Adherence to FeSS Protocols and treatment on a stroke unit

Patients that received treatment in stroke units had double the odds of receiving care in accordance with FeSS Protocols (aOR 2.3, 95 %CI: 2.0, 2.5, *p* < 0.001), compared to those who were not treated in a stroke unit regardless of the time period (Table 2). Similar levels of improvement over time was found for those treated on a stroke unit and for those treated outside of a stroke unit (pre-guideline: stroke unit care 40 %, non-stroke unit care 21 %, post-guideline: stroke unit care 44 %, non-stroke unit care 27 %, *p*-value for interaction term *p* = 0.07). (Supplemental Table I). Results of the sensitivity analyses comparing 2017 and 2021 Audit cycles were consistent with the main results presented (Supplemental Table II).

### 3.5 Adherence to individual FeSS indicators post-inclusion in Stroke Guidelines

Comparison of adherence to the individual FeSS indicators pre -and post- guideline was variable (Table 3). There was no significant difference from pre-to post-guidelines in the increased proportion of patients who received prompt treatment with paracetamol for fever (pre-guideline: 53 %, post-guideline: 50 %). However, swallow screen within 24 hrs (pre-guideline: 68 %, post-guideline: 75 %; *p* < 0.001); swallow screen or assessment before oral food or fluids (pre-guideline: 60 %, post-guideline: 65 %; *p* < 0.001) and before oral medications (pre-guideline: 55 %, post-guideline: 60 %; *p* < 0.001) did show significant improvements. Prompt treatment with insulin for blood glucose level (BGL) >10 mmol/l post-Stroke Guideline inclusion was significantly lower (pre-guideline: 34 %, post- guideline: 30 %; *p* = 0.041) (Table 3).

**Table 3:** Adherence to FeSS Protocols composite measure and individual FeSS indicators by pre-post guideline.

	Pre- Guideline (*N* = 7011)	Post- Guideline (*N* = 7195)	aOR (95 % CI)^a^	*p*-value
Adherence to FeSS Protocols composite measure	2449 (35 %)	2864 (40 %)	1.2 (1.2, 1.3)	**<0.001**
1. Patient developed fever ≥ 37.5 °C within first 72 h	778 (11 %)	760 (11 %)	0.94 (0.85, 1.1)	0.315
2. Paracetamol for the first elevated temperature administered within 1 h	342 (53 %)^b^	337 (50 %)^b^	0.84 (0.66, 1.1)	0.147
3. Hyperglycaemia (first 48 h of admission)	1211 (20 %)	1336 (20 %)	1.0 (0.95, 1.1)	0.452
4. Insulin administered within 1 hour of the first elevated finger-prick glucose (>10 mmol/L)	412 (34 %)	404 (30 %)	0.83 (0.69. 0.99)	**0.041**
5. Swallow screen or assessment within 24hrs	4000 (68 %)^c^	4433 (75 %)^c^	1.4 (1.3, 1.5)	**<0.001**
6. Swallow screen or assessment before oral food or fluids	4203 (60 %)	4648 (65 %)	1.2 (1.1, 1.3)	**<0.001**
7. Swallow screen or assessment before oral meds	3882 (55 %)	4346 (60 %)	1.2 (1.2, 1.3)	**<0.001**
8. Failed swallow screen and referred to Speech Pathologist	1404 (97 %)	1562 (96 %)	0.81 (0.42, 1.6)	0.524

Not documented considered No, and included in denominator; aOR - adjusted odds ratio, CI – confidence interval.

a adjusted for stroke unit care, hospital prior participation in treatment arm of a FeSS Intervention study, age, sex, stroke type, stroke severity, including correlation of outcomes within hospital;.

b Excludes those already receiving regular paracetamol or where contraindicated.

c Only cases with valid times included.

### 3.6 Individual FeSS indicators compared to previous FeSS Intervention studies

Adherence to each of the individual FeSS indicators from the pre- and post- guideline Audit data was informally compared to the post-intervention results from the FeSS Intervention studies (QASC 2010, QASCIP 2014, T^3^ 2016) (Table 4). The overall proportion of patients who were treated with paracetamol for fever, and insulin for hyperglycaemia, has improved in clinical practice both before and after the guideline recommendation when compared to each of the FeSS Intervention studies. In contrast, adherence to the swallowing variables was generally better in the FeSS Intervention studies. In the original QASC study, the proportion of patients who received a swallow screen or assessment within 24 h (81 %) was greater than either of the Audit groups (pre-guideline (68 %), post-guideline (75 %). Higher proportions of adherence to all swallowing variables were reported in the most recent FeSS Intervention study (T^3^) compared to both Audit groups. This was also true for three of the four swallowing variables in the QASCIP study, with the exception of the proportion of patients who received a swallow screen or assessment within 24 h which was reported to be the same as post-guideline Audit data. We were unable to directly compare the composite measures from all the FeSS Intervention studies as these included monitoring variables for fever and hyperglycaemia which are no longer collected in the Audits after 2015.

**Table 4:** Post-intervention results for individual FeSS Intervention studies compared to pre-and post- Stroke Guideline inclusion Audit data.

	QASC^a^ (2010)	QASCIP^a^ (2014)	T^3a^ (2016)	QASC Europe^a^^,^^c^ (2021)	Pre- guideline (2015, 2017 Audit)	Post- guideline (2019, 2021 Audit)
Fever (≥37.5 °C)	17 %	12 %	4 %	18 %	11 %	11 %
Paracetamol within 1 h for fever	18 %	47 %	6 %	79 %	53 %	50 %
Hyperglycaemia (>10 mmol/L)	20 %^b^	19 %	13 %	21 %	20 %	20 %
Insulin within 1 hour for hyperglycaemia	14 %	27 %	10 %	75 %	34 %	30 %
Swallow screen/ assessment within 24 h	81 %	75 %	81 %	79 %	68 %	75 %
Swallow screen/assessment before oral food or fluid	22 %	68 %	90 %	82 %	60 %	65 %
Swallow screen/assessment before oral medications	37 %	62 %	75 %	82 %	54 %	59 %
Swallow assessment if failed screen	78 %	95 %	91 %	66 %	86 %	87 %

a Reference is post-intervention results.

b >11 mmol/L in QASC Trial.

c QASC Europe 2017–2021 (Pre-Post International Study: Supported implementation FeSS Protocols in stroke services) ^28^ QASC 2005–2010 (Randomised Controlled Trial: Supported implementation FeSS Protocols in stroke units) ^1^ QASCIP 2013–2014 (Pre-Post Study: Supported implementation FeSS Protocols in all stroke services NSW) ^3^ T^3^ 2013–2016 (Randomised Controlled Trial: Supported implementation FeSS Protocols in Emergency Departments)^4^.

## 4 Discussion

Overall, the findings of this study report two in five Australian stroke patients are being cared for in accordance with FeSS Protocols, despite a strong recommendation for their use in the Stroke Guidelines in 2017. There has been improvement in adherence to FeSS Protocols since this recommendation was made however, the higher adherence to FeSS Protocols pre-guideline for those hospitals that did participate in any treatment arm of the FeSS Intervention studies (QASC (2005–2010), QASCIP (2013–2014) or T^3^ (2013–2016) suggests that involvement in implementation research studies can enhance adherence to recommended care. Although there has been some improvement in adherence in non-stroke unit settings, patients treated in stroke units have double the odds of receiving care in accordance with FeSS Protocols.

Clinical practice guidelines are an essential source of current evidence-based recommendations for clinicians. Including research into national guidelines, although an accomplishment in itself, does not guarantee adherence. In the United States of America, the United Kingdom (UK) and Australia, patients receiving care according to clinical practice guidelines is widely acknowledged to be approximately 60 % (Braithwaite et al., 2020). Guideline dissemination is generally considered to be a ‘passive’ implementation strategy when compared to more ‘active’ and often multi-faceted implementation strategies used in implementation research trials (Vedel et al., 2018). These varied approaches to getting evidence into practice (health professionals’ behaviour change) has been the subject of much research over the decades (Grimshaw et al., 2006; Bero et al., 1998; Grimshaw et al., 2001). With a growing body of literature demonstrating a positive association between the number of evidence-based care processes received in hospital and patient outcomes (Middleton et al., 2019; Muñoz Venturelli et al., 2019; Cadilhac et al., 2017) it is vital that future research is directed towards guideline adoption.

The improvement in adherence to FeSS Protocols is not possible to attribute solely to inclusion of the new guideline recommendation. Although the promotion of this new recommendation may have contributed to the overall secular trend, it may also be due to participation in the Audit cycles itself. Audit and feedback is reported to have a modest effect size (median 4.3 %); however, the range is widely variable (0.5–16 %), dependent upon baseline performance and the conditions for feedback (Stroke Foundation, 2018).

Coinciding with the new guideline recommendation for use of the FeSS Protocols in 2017, was the Stroke Foundation's partnership with Cochrane Australia to develop the world's first living Clinical Guidelines for Stroke Management in 2018 (Stroke Foundation 2018). This dynamic model ensures new evidence relevant to the guideline topics is continually monitored and stroke evidence is updated as required to guide clinical practice and policy development (English et al., 2022). Although a recent evaluation of the introduction of this living Stroke Guidelines model reported a threefold increase in online access since their inception (Wiles et al., 2024) this is not indicative of active organisational implementation efforts (Straus et al., 2013).

Previous participation in any treatment arm of the FeSS Intervention studies suggests that exposure to the multi-faceted implementation strategies used in these studies (audit and feedback, clinical champions, barrier and enabler assessments, educational workshops and reminders) (Middleton et al., 2011, 2016); provided greater implementation outcomes that are sustained long after the clinical trial is completed. All hospitals that participated in the QASC Trial were included in the subsequent FeSS Intervention study QASCIP, irrespective of the treatment group. This was essentially an additional ‘dose’ of the intervention for those hospitals in the intervention arm of the QASC Trial and/or likewise if they were in the intervention arm of the T^3^ Trial (Fig. 1). Given the small numbers, different study designs and timing of the interventions in these studies, any further statistical inferences about the potential added benefits of multiple study participation would be unreliable and require cautious interpretation. This repeated exposure to the FeSS Protocols and the implementation strategies may explain why our findings support those first reported by Purvis et al. (2019) who found that previous participation in a FeSS Intervention study was associated with improved adherence to the FeSS Protocols (Purvis et al., 2019). This is in contrast to the literature in relation to ‘voltage drop’ and ‘program drift’ that assumes the effect of an intervention decreases after a clinical trial as it moves into the dissemination and implementation research stages (Chambers et al., 2013; Nadalin Penno et al., 2019). Some decay effect is to be expected when assessing sustainability longer term (Cadilhac et al., 2017), however maintenance of and continued adherence to FeSS Protocols suggests that the external support (e.g. training, resources, performance feedback) (Middleton et al., 2011, 2016, 2019) to facilitate organizations to adopt these practices have been sustainable longer term (Francis et al., 2016). Although there was not an increase in the amount of change over time when compared to hospitals that did not participate in these studies (or were in the control arm), being in the treatment arm of any of the FeSS Intervention studies did result in a higher level of adherence pre-guideline that was upheld over time.

In general, research active hospitals produce better patient outcomes and quality of care (Australian Academy of Health and Medical Sciences, 2022; Purvis et al., 2016). This may explain why those health services that elected to participate in the FeSS Intervention studies were more likely to adhere to clinical practice guidelines (Stroke Foundation, 2021). The hospitals that participate in the Audits may likely also be quality-improvement/ research ‘active’ with high voluntary participation rates reported each Audit cycle (Stroke Foundation, 2019, 2021) that suggest high motivation to improve care.

The evidence for stroke unit care and better patient outcomes is well established (Langhorne P DM, 1998), yet the exact reasons why stroke units have this effect are still yet to be defined (Langhorne and Ramachandra, 2020). The specialist multidisciplinary care and proactive prevention of common post stroke complications are among the many hypotheses for stroke unit effectiveness (Langhorne P DM, 1998). Our study findings demonstrate adherence to FeSS Protocols is markedly lower for patients that do not receive treatment in a stroke unit. Ideally, every patient with stroke should be able to access stroke unit care however, this is not always practical in remote areas or smaller hospitals. We have previously reported that the FeSS Protocols can be implemented in non-stroke unit settings (Ding et al., 2024). Understanding the barriers to implementation in these settings requires further investigation.

The FeSS Protocols and resources to implement have been freely available on the QASC research program website (Australian Catholic University, 2024) since the publication of the QASC trial results in 2011. Despite the concerted dissemination efforts of the trial results and inclusion in the national clinical guidelines for stroke management, the use of FeSS Protocols has not translated into everyday clinical practice for all Australian stroke patients.

The plateau in the uptake of FeSS Protocols and wide variation in practice is not unique to this stroke clinical practice guideline recommendation or country (Middleton et al., 2019; Stroke Foundation, 2021; Levi et al., 2020; European Stroke Initiative Executive Committee et al., 2003; Adams et al., 2007; Intercollegiate Stroke Working Party, 2012). The supported implementation of FeSS Protocols by our team in the QASC Europe Study (2017–2021) (Middleton and McElduff, 2022) conducted in 64 hospitals in 17 countries, reported a very low baseline level of adherence to FeSS Protocols (3.4 %) and whilst, following our facilitated implementation, this improved to 35 % with an absolute difference of 33 %, (95 % CI: 24 %, 42 %), again, there is room for improved compliance. Of note, however, this more recent international comparison demonstrates higher levels of adherence (especially with prompt paracetamol or insulin administration) than previously achieved in any Australian FeSS Intervention study suggesting that improvement in these areas are possible.

The most recent Cochrane systematic reviews to address the effectiveness of interventions for the uptake of evidence‐based recommendations in both acute stroke settings (Lynch et al., 2023) and stroke rehabilitations services (Cahill et al., 2020) cited the small number of studies as the reason they were unable to determine if multi-faceted implementation strategies were more effective than no intervention. Our study adds to their call for additional research that evaluates the implementation of evidence-based clinical practice guidelines and strategies that support this.

Limitations related to this study were the retrospective design of the Audits and reliance on a one-time snapshot of approximately 40 cases per hospital at each cycle which may reduce the generalizability of the findings. The lack of randomization, voluntary participation and self-report is subject to reporting and/or response bias and some of the audit questions have changed over the cycle years which meant we were only able to include those variables that were collected in all cycles (e.g. risk factors for stroke).

The reliability of documentation in medical records and the assumption that if a process was not documented, it was not performed, needs to be acknowledged. In this study, older patients (≥ 85 years) had greater odds of receiving treatment in accordance with FeSS Protocols possibly related to the likelihood of co-morbidities that required closer monitoring. However, patients with haemorrhagic stroke had significantly lower odds of being treated according to the FeSS Protocols even though this stroke subtype is generally considered to be the most severe with the highest morbidity and mortality rates (Woo et al., 2022).

Although we excluded any patients who had been documented for palliative care, we cannot be sure that some of the haemorrhagic stroke admissions were missing this important documentation and were, in fact, being treated with palliative care measures.

Similarly, for those patients who presented with mild stroke severity, the working diagnosis on admission may have been TIA which then progressed to stroke after further investigations and assessment during the course of their episode of care. Again, relying on documentation in the medical record may not capture this development accurately. However, we do know that it is common for this mild stroke population to be undertreated (Finch et al., 2017; Gong et al., 2022) and discharged with unmet needs (Finch et al., 2017) so this could also be yet another example of undertreatment.

The strengths of using the Audit data include the large sample size that is able to provide a robust and reliable overview of a cross-sectional snapshot of Australian acute stroke services and adherence to stroke clinical guidelines. Participation rates are high, 89 % of acute admissions for stroke are to hospitals that participate in the Audit (Stroke Foundation, 2019; AIHW, 2021) (comparable to 90 % of admitted strokes in the UK mandatory stroke audit program) (SSNAP, 2023).

The use of this large dataset provides a more representative view of the wider Australian stroke population that includes both stroke unit and non-stroke unit care. As a measure of sustainability, the use of these data provide an estimate for the level of diffusion (Francis et al., 2016) for use of the FeSS Protocols outside of these specialist stroke units. Standardised training for data abstraction are provided for each Audit cycle and reliability cases are also reported which increase the confidence in use of this large dataset (Stroke Foundation, 2020).

Use of a composite outcome to assess adherence to the FeSS Protocols can be subject to limitations that relate to how missing data is handled, and the weighting of the individual FeSS indicators that make up this measure. To ensure consistency, missing data were handled in alignment with previous FeSS adherence research (Middleton et al., 2011, 2016, 2015; Purvis et al., 2019; Middleton and McElduff, 2022).

Although the individual components that make up the composite measure are all related as care processes within the FeSS Protocols, the choice of these components was subject to ‘availability bias’ (Barclay et al., 2019) and the measures that were captured in the Stroke Foundation Audits. The Audits capture fever and hyperglycaemia treatment but not monitoring variables (which are collected in the FeSS Intervention studies). Unfortunately, the nature of using a binary composite outcome can emphasise the process of care the patient failed to receive, without acknowledging that they may have received all other components that make up this composite measure. The bundled nature of the FeSS Protocols means that it is not possible to separate these processes out, as one being more important than the other, as we do not have the evidence to do so. However, we have presented the individual components that make up this measure to provide transparency in relation to where any overall shortcomings may be. These results suggest that the improvement in swallowing management may have driven the increase in overall adherence to FeSS Protocols. The simplicity of this outcome measure does provide an overview that enables appropriate comparison with previous work and composite outcomes that have been used throughout this research program.

## 5 Conclusions

This study provides new insights into the evaluation of the natural history and longer-term results of diffusion, dissemination, and implementation of stroke clinical practice guidelines in Australian acute care hospitals. It has now been over a decade since the seminal QASC trial results were published and six years since the inclusion of a strong recommendation for their use in the Stroke Guidelines. Overall, adherence to FeSS Protocols has increased with a recommendation for their use in the guidelines (and audit and feedback associated with the Audits). However, with only two in five patients receiving this care, further efforts are still required to encourage more widespread adoption. As expected, stroke unit care was associated with increased adherence to FeSS Protocols that was sustained over time. The improvement in adherence to this clinical practice guideline was also increased and sustained over time for hospitals with previous exposure to the FeSS Protocols in any of the FeSS Intervention studies. This suggests that exposure to the more active implementation strategies and involvement in implementation research studies can enhance adherence to recommended care.

The significant costs and resources associated with these strategies could be offset by reserving these efforts for a more targeted delivery approach, where variation in practice has been identified. Future research is required in relation to how these sites are identified, and the levels of intervention required (Fasugba et al., 2023) to improve uptake in use of the FeSS Protocols.

## Declarations

Availability of data and materials

The data that support the findings of this study are available from the Stroke Foundation (Acute Audits) but restrictions apply to the availability of these data which were used under contract for the current study, and so are not publicly available. Data may be available from the authors upon reasonable request and with permission of the Stroke Foundation (Acute Audits).

## Competing interests

KH (National Manager Stroke treatment, Stroke Foundation)

MK, MR, TP and DC are responsible for the independent and alloy analysis of National Stroke Audit data on behalf of the Stroke Foundation

MK (member of the Research Advisory Committee at the Stroke Foundation)

SM, CL, DAC, SD, NWC, JG, CDE, EM, KC, OF, BM (Investigators QASC Research Program: QASC QASCIP,T^3^ and QASC Europe Studies)

## Funding

This study was funded by a National Health and Medical Research Council Investigator Grant (Grant ID: APP1196352) awarded to SM. It is also supported by a small grant from St. Vincent's Hospital Melbourne Research Endowment Fund (94,653). KC is also supported by an Australian Government Research Training Program Scholarship. MFK is supported by 10.13039/501100001030National Heart Foundation of Australia fellowship (105,737). The funding bodies have no role in the design of the study and collection, analysis, and interpretation of data and in writing the manuscript.

## CRediT authorship contribution statement

**Kelly Coughlan:** Conceptualization, Methodology, Software, Formal analysis, Data curation, Writing – original draft, Writing – review & editing, Visualization, Project administration, Funding acquisition. **Tara Purvis:** Conceptualization, Methodology, Software, Formal analysis, Resources, Data curation, Visualization, Writing – review & editing. **Monique F. Kilkenny:** Conceptualization, Methodology, Software, Formal analysis, Resources, Data curation, Visualization, Writing – review & editing. **Dominique A. Cadilhac:** Methodology, Resources, Writing – review & editing. **Oyebola Fasugba:** Conceptualization, Methodology, Visualization, Writing – review & editing, Supervision. **Simeon Dale:** Conceptualization, Methodology, Visualization, Writing – review & editing, Supervision. **Kelvin Hill:** Conceptualization, Methodology, Resources, Writing – review & editing, Supervision. **Megan Reyneke:** Resources, Writing – review & editing. **Elizabeth McInnes:** Conceptualization, Writing – review & editing, Supervision. **Benjamin McElduff:** Conceptualization, Methodology, Software, Formal analysis, Data curation, Writing – review & editing. **Jeremy M. Grimshaw:** Writing – review & editing. **N Wah Cheung:** Writing – review & editing. **Christopher Levi:** Writing – review & editing. **Catherine D'Este:** Conceptualization, Methodology, Software, Formal analysis, Data curation, Writing – review & editing, Visualization. **Sandy Middleton:** Conceptualization, Methodology, Resources, Writing – original draft, Writing – review & editing, Visualization, Supervision, Funding acquisition.

## Declaration of competing interest

The authors declare the following financial interests/personal relationships which may be considered as potential competing interests:

Professor Sandy Middleton reports financial support was provided by National Health and Medical Research Council Investigator Grant. Kelly Coughlan reports financial support was provided by Australian Government Research Training Program Scholarship. Monique Kilkenny reports financial support was provided by National Heart Foundation of Australia fellowship. Kelly Coughlan reports financial support was provided by St Vincent's Hospital (Melbourne) Limited. Kelvin Hill reports a relationship with Stroke Foundation Australia that includes: employment. Co-author Monique Kilkenny is a member of the Research Advisory Committee at Stroke Foundation Australia Co-authors Monique Kilkenny, Megan Reyneke, Tara Purvis and Dominique Cadilhac are responsible for the independent and alloy analysis of National Stroke Audit data on behalf of the Stroke Foundation Corresponding author and co-authors Sandy Middleton, Christopher Levi, Dominique Cadilhac, Simeon Dale, N Wah Cheung, Jeremy Grimshaw, Cate DEste, Elizabeth McInnes, Kelly Coughlan, Oyebola Fasugba, are investigators for the QASC Research Program (QASC QASCIP,T3 and QASC Europe Studies)

If there are other authors, they declare that they have no known competing financial interests or personal relationships that could have appeared to influence the work reported in this paper.

## Appendix Supplementary materials

Image, application 1
