# Supplementary material for: From ‘strong recommendation’ to practice: A pre-test post-test study examining adherence to stroke guidelines for fever, hyperglycaemia, and swallowing (FeSS) management post-stroke
Source: Int J Nurs Stud Adv. 2024 Oct 23;7:100248. doi: 10.1016/j.ijnsa.2024.100248 (PMC11539718; doi:10.1016/j.ijnsa.2024.100248)
Supplement: Supplementary file 1 [file mmc1.docx]

**Supplemental Table I: Change in adherence to FeSS Protocols composite measure pre-post-guideline by i) prior participation in any treatment arm of a FeSS Intervention study^#^ and ii) treatment in a stroke unit**

|  | **Pre-guideline**  **N=7011** | **Post-guideline**  **N=7195** | ***p*-value**^ab^ |
| --- | --- | --- | --- |
| Hospital prior participation in any treatment arm of a FeSS Intervention study^c^ | 40% | 46% | 0.93 |
| No prior participation in a FeSS Intervention study (or in control arm) | 31% | 35% |  |
| ^a^ p-value of interaction, ^b^adjusted for stroke unit care, age, sex, stroke type, stroke severity, including correlation of outcomes within hospital;  ^c^ Intervention arm QASC 2005-2010 (Randomised Controlled Trial: Supported implementation FeSS Protocols in stroke units) ^1^  All hospitals QASCIP 2013-2014 (Pre-Post Study: Supported implementation FeSS Protocols in all stroke services NSW) ^3^  Intervention arm T^3^ 2013-2016 (Randomised Controlled Trial: Supported implementation FeSS Protocols in Emergency Departments)^4^ | | | |
|  | **Pre-guideline**  **N=7011** | **Post-guideline**  **N=7195** | ***p*-value** ^ab^ |
| Treatment in a stroke unit | 40% | 44% | 0.07 |
| Treatment outside of a stroke unit | 21% | 27% |  |
| ^a^ p-value of interaction, ^b^adjusted for hospital prior participation in treatment arm of a FeSS Intervention study, age, sex, stroke type, stroke severity including correlation of outcomes within hospital; | | | |

**Supplemental Table II: Sensitivity analysis: Audit cycle 2017 compared to Audit cycle 2021 for adherence to FeSS Protocols composite outcome**

|  | **aOR**^b^ | **95% CI** | ***p*-value** |
| --- | --- | --- | --- |
| 2021 Audit cycle^a^ | 1.1 | 1.0, 1.3 | **0.016** |
| Hospital participated/in treatment arm FeSS Intervention study | 1.7 | 1.3, 2.1 | **<0.001** |
| Treatment in a stroke unit | 2.2 | 1.9, 2.5 | **<0.001** |
| Age |  |  |  |
| <65 y | ref |  |  |
| 65-74 y | 1.1 | 0.9, 1.3 | 0.304 |
| 75-84 y | 1.1 | 0.9, 1.3 | 0.270 |
| ≥85 y | 1.2 | 1.1, 1.4 | **0.012** |
| Male | 1.0 | 0.9, 1.1 | 0.739 |
| Haemorrhagic stroke | 0.75 | 0.6, 0.9 | **0.001** |
| Walk independently on admission | 0.78 | 0.7, 0.9 | **<0.001** |

^a^Reference is 2017 Audit cycle; ^b^adjusted for factors listed in table, including correlation of outcomes within hospital;

aOR - adjusted odds ratio, CI – confidence interval
